# Factors associated with quitting status of smoking in Korean men with and without chronic kidney disease: A national population-based study

**DOI:** 10.18332/tid/145698

**Published:** 2022-02-17

**Authors:** Youngmee Kim, Won-Kyung Cho

**Affiliations:** 1 Red Cross College of Nursing, Chung-Ang University, Seoul, Republic of Korea; 2 Department of Pulmonology and Critical Care Medicine, Asan Medical Center, University of Ulsan, Seoul, Republic of Korea; 3 International Healthcare Center, Asan Medical Center, University of Ulsan, Seoul, Republic of Korea

**Keywords:** chronic kidney disease, CKD, Koreans, male smokers, smoking cessation

## Abstract

**INTRODUCTION:**

Adverse effects of smoking on kidney function have been demonstrated in both general populations and in populations with chronic kidney disease (CKD). Therefore, quitting smoking can have a significant impact on the mortality and disease progression of CKD. This study examined and compared factors associated with quitting status of smoking, in patients with and without CKD, among Korean adult male smokers, using the Korea National Health and Nutrition Examination Survey from 2008 to 2019, excluding 2013.

**METHODS:**

Wald test with multiple logistic regression was performed to investigate factors associated with quitting smoking in both CKD and non-CKD groups, along with the interaction effects between groups.

**RESULTS:**

Of the 15747 eligible individuals, 909 had CKD, of whom 703 (weighted percentage: 74.4%) were quitters. In the non-CKD group, 8393 (weighted percentage: 50.4%) succeeded in quitting. Regular exercise was the only factor associated with quitting in both groups. The adjusted odd ratios with confidence intervals were 1.29 (95% CI: 1.17–1.42) and 2.84 (95% CI: 1.52–5.31) in the non-CKD and CKD groups, respectively (interaction p=0.0153). Unlike in the CKD group, marriage and higher systolic blood pressure were also associated with quitting, and lifetime smoking amount and secondhand smoke exposure at home were negatively associated with smoking cessation in the non-CKD group.

**CONCLUSIONS:**

Exercise was the only factor associated with quitting smoking in the CKD group.

## INTRODUCTION

Chronic kidney disease (CKD) is defined as kidney damage or decreased kidney function for more than three months^1^. It is estimated that about 9% of the population is affected by CKD worldwide. In 2017, CKD resulted in 1.2 million deaths and was the 12th leading cause of death worldwide. The significant causes of death in the CKD population are disease progression or CKD-attributable cardiovascular disease (CVD)^2^. High cardiovascular mortality in this population can be partly explained by the frequent occurrence of CKD in diseases associated with cardiovascular risk, such as hypertension and diabetes. However, CKD is also an independent risk factor for cardiovascular death^1,3,4^. Smoking tobacco is a leading cause of CVD morbidity and mortality^5^. This means that patients with CKD, who already have a higher CVD risk than the general population, are at an even greater risk if they smoke.

CKD may progress to end-stage kidney disease. Some patients with CKD demonstrate a more rapid progression rate, while others have relatively stable disease. Various factors have been suggested as contributors to disease progression in patients with CKD or modifiers of the disease course, such as comorbid conditions, treatments, socioeconomic status, and ethnicity^6-8^. Adverse effects of smoking on kidney function have been demonstrated in both the general population and CKD-specific populations. Specifically, it has been reported that albuminuria, nephrosclerosis or glomerulonephritis are more common in smokers^8-15^. The mechanisms of smoking-related kidney damage can be explained by hemodynamic or non-hemodynamic effects, such as nicotine toxicity, oxidative stress, thrombotic, and inflammatory cytokine release^9^. This means that smoking can modify the disease course of CKD. Indeed, a few studies have suggested that smoking cessation may slow CKD progression^9,16,17^. These findings indicate that quitting smoking can significantly impact the mortality and disease progression of CKD. Many smokers, aware of tobacco’s harmfulness, want to quit but often fail. Considering how difficult it is to quit smoking, identifying the factors involved is the first step in helping people to do so.

Numerous physiological, behavioral, environmental, psychological, cognitive, and social factors are involved in the success or failure of smoking cessation in the general population^18-21^. However, there have been no studies to examine CKD-specific factors related to smoking cessation. In this context, this study examined and compared the quitting status of smoking and the factors associated with quitting status in study participants with and without CKD among Korean adult male smokers using nationally representative data, the Korea National Health and Nutrition Examination Survey (KNHANES).

## METHODS

### Study design and ethical considerations

This study was conducted using data from the Korea National Health and Nutrition Examination Survey (KNHANES) from 2008 to 2019, excluding 2013 because it did not contain detailed information on smoking cessation. The KNHANES is an ongoing, annual, nationwide, population-based, cross-sectional, multistage, stratified, and clustered probability sampling survey based on geographical area, age, and gender by the Korea Centers for Disease Control and Prevention (KCDC), which examines the health, lifestyle, and eating habits of Koreans^22^. The data consist of questionnaires on physical and mental health and laboratory tests. KNHANES consists of three components: a health interview, health examination, and nutrition survey. The health interview and health examination are conducted by trained medical staff and interviewers at the mobile examination center. A week after the health interview and examination surveys, dieticians visit the participants’ homes for the nutrition survey. Also, blood samples are collected at the mobile examination centers in the morning after a fast by participants of at least 8 hours for the collection of laboratory data.

The data were downloaded after the registration process designated for accessing the official KNHANES website (http://knhanes.cdc.go.kr/). Before the survey, written informed consent was obtained from each study participant^22^. The current study used only existing de-identified data. The study was conducted according to the guidelines of the Declaration of Helsinki. The institutional review board (IRB) of the Korea Centers for Disease Control and Prevention (KCDC) reviewed and approved the KNHANES survey. The IRB approval numbers were 2008-04EXP-01-C, 2009-01CON-03-2C, 2010-02CON-21-C, 2011-02CON-06-C, 2012-01EXP-01-2C, 2013-12EXP-03-5C, 2018-01-03-P-A, and 2018-01-03-C-A.

### Participants

The inclusion criteria of the present study were men aged ≥19 years having serum creatinine results, who have smoked more than 100 cigarettes in their lifetime, and have tried to quit smoking in the past. We excluded: 1) never smokers or persons with a lifetime smoking history of <100 cigarettes; and 2) those who did not attempt to quit smoking. A total of 93120 people were screened, and finally, 15747 people were included in the study, according to the inclusion criteria. The final sample comprised 14838 men without CKD and 909 men with CKD.

This study included only men because smoking history was examined using only self-report data. Previous studies showed a significant difference between self-reported smoking rates and those assessed by measurement of cotinine level in Korean women, which is thought to be related to social stigma and prejudice they face^23-25^. Due to the limited availability of cotinine levels in the KNHANES data, we have decided to include only men in the analysis.

**Figure 1:**
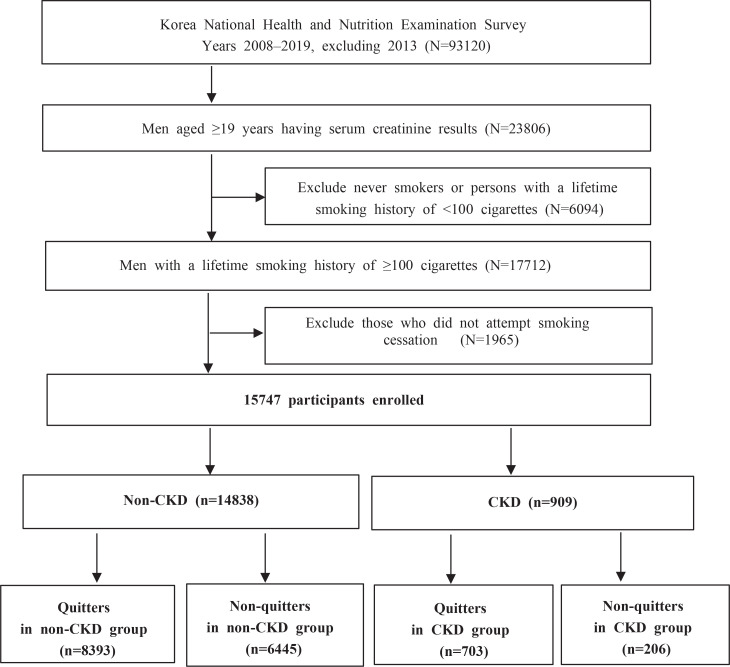
Flow diagram showing participant selection and the numbers of participants

Figure 1 demonstrates the flow diagram of the selection process and the number of study participants.

### Defining and measuring important variables

In this study, CKD was defined as an estimated glomerular filtration rate (eGFR) <60 mL/min/1.73 m^2^. The eGFR was calculated using the Modification of Diet in Renal Disease equation^26,27^.

Quitters were defined as those who reported having smoked more than 100 cigarettes in their lifetime and who were no longer smokers at the time of the survey. Non-quitters were those who reported having smoked more than 100 cigarettes in their lifetime and were smokers at the time of the study, with unsuccessful past attempts to quit smoking^28,29^. Our definitions of smoker, non-smoker, quitter, and non-quitter, are based on the CDC recommendations^28,29^.

Other than current smoking status, additional smoking-related histories such as lifetime smoking amount (pack-years), exposure to secondhand smoke, and smoking cessation methods were obtained using a questionnaire. Regular exercise was defined as exercising at a vigorous intensity for 20 or more minutes per session at least three times a week or exercising at moderate intensity for 30 or more minutes per session at least five times a week, performing vigorous exercise for 1.25 hours or more a week, or a moderate level of exercise for 2.5 hours or more a week. Participants were advised to answer exercise intensity according to the subjective degree of body’s hardship and shortness of breath. To help participants better understand the categories, some examples were also provided in the questionnaire. For example, running, swimming, and lifting heavy objects are regarded as vigorous exercises. Table tennis and lifting light objects are considered moderate exercises.

Trauma history was defined as a history of at least one accident or intoxication that required hospitalization and/or emergency room treatment during the past year. Heavy drinkers were defined as those who consume seven or more glasses of alcohol (regardless of alcohol type) per occasion at least twice per week. Perceived health status was defined as an individual’s perceived overall health level (i.e. very good/good, fair, or poor/very poor). Perceived psychological stress was defined as moderate-to-severe daily stress^22,30^.

### Statistical analysis

All data analyses were conducted using SAS version 9.4 (SAS Institute, Inc., Cary, NC, USA) and presented as mean ± standard error (SE) for continuous variables or as proportions (±SE) for categorical variables. The prevalence of quitters and non-quitters according to the absence and presence of CKD was presented as a proportion (±SE) (Table 1). T-tests and chi-squared tests were conducted to evaluate the differences between groups for continuous and categorical variables, respectively (Tables 2 and 3).

**Table 1:** Prevalence of quitters and non-quitters according to non-CKD and/or CKD groups (N=15747)

*Year*	*Total (N=15747)*			*Non-CKD (n=14838)*			*CKD (n=909)*		
	*Quitter (n=9096)^a^ % (SE)*	*Non-quitter (n=6651)^b^ % (SE)*	*p*	*Quitter (n=8393)^c^ % (SE)*	*Non-quitter (n=6445)^d^ % (SE)*	*p*	*Quitter (n=703)^e^ % (SE)*	*Non-quitter (n=206)^f^ % (SE)*	*p*
2008	48.7 (1.47)	51.3 (1.47)	<0.0001	47.3 (1.50)	52.7 (1.50)	<0.0001	75.4 (5.31)	24.6 (5.31)	0.0309
2009	46.1 (1.35)	53.9 (1.35)		45.3 (1.37)	54.7 (1.37)		75.7 (6.03)	24.3 (6.03)	
2010	46.3 (1.76)	53.7 (1.76)		45.3 (1.81)	54.7 (1.81)		78.0 (5.88)	22.0 (5.88)	
2011	47.9 (1.68)	52.1 (1.68)		46.4 (1.71)	53.6 (1.71)		81.0 (5.90)	19.0 (5.90)	
2012	50.6 (1.61)	49.4 (1.61)		50.3 (1.64)	49.7 (1.64)		60.4 (7.75)	39.6 (7.75)	
2014	47.6 (2.04)	52.4 (2.04)		46.9 (2.07)	53.1 (2.07)		70.6 (7.49)	29.4 (7.49)	
2015	54.7 (1.51)	45.3 (1.51)		53.7 (1.58)	46.3 (1.58)		78.1 (5.46)	21.9 (5.46)	
2016	55.0 (1.79)	45.0 (1.79)		53.5 (1.85)	46.5 (1.85)		88.7 (3.38)	11.3 (3.38)	
2017	57.4 (1.68)	42.6 (1.68)		56.9 (1.73)	43.1 (1.73)		69.5 (5.90)	30.5 (5.90)	
2018	59.3 (1.50)	40.7 (1.50)		58.6 (1.56)	41.4 (1.56)		79.4 (5.35)	20.6 (5.35)	
2019	59.1 (1.48)	40.9 (1.48)		58.8 (1.52)	41.2 (1.52)		63.4 (5.93)	36.6 (5.93)	

Data have been presented as weighted percentages (standard error, SE). The p-value was determined using the Rao-Scott chi-squared test. Data were not collected in 2013.

a Weighted n (weighted %)=6214858 (51.3%).

b Weighted n=5895954 (48.7%).

c Weighted n=5876685 (50.4%).

d Weighted n=5779842 (49.6%).

e Weighted n=338173 (74.4%).

f Weighted n=116112 (25.6%).

CKD: chronic kidney disease.

**Table 2:** Sociodemographic characteristics and smoking history of participants (N=15747)

*Variables*	*Non-CKD (n=14838)*			*CKD (n=909)*		
	*Quitter (n=8393) % (SE)*	*Non-quitter (n=6445) % (SE)*	*p*	*Quitter (n=703) % (SE)*	*Non-quitter (n=206) % (SE)*	*p*
**Age** (years), mean ± SE	51.65 ± 0.20	42.08 ± 0.21	<0.0001	67.72 ± 0.58	61.78 ± 1.16	<0.0001
**Age** (years)			<0.0001			<0.0001
19–39	21.6 (0.63)	46.3 (0.81)		1.4 (0.75)	3.8 (2.27)	
40–64	57.2 (0.67)	47.5 (0.78)		32.0 (2.33)	53.7 (4.38)	
≥65	21.2 (0.51)	6.2 (0.28)		66.7 (2.35)	42.5 (4.18)	
**Married**	84.3 (0.52)	65.4 (0.80)	<0.0001	84.8 (1.82)	81.4 (3.62)	0.3709
**Education level**			0.4447			0.1446
≤High school	60.1 (0.78)	60.8 (0.77)		80.2 (1.94)	73.7 (4.30)	
University or higher	39.9 (0.78)	39.2 (0.77)		19.8 (1.94)	26.3 (4.30)	
**Occupation**			<0.0001			0.0009
Manager/professionals	16.7 (0.57)	16.8 (0.57)		5.9 (1.32)	17.1 (3.96)	
Office worker	12.7 (0.46)	12.9 (0.50)		4.9 (1.13)	2.5 (1.10)	
Service workers/sellers	10.9 (0.44)	14.8 (0.56)		5.6 (1.21)	8.9 (2.86)	
Agriculture/fishery/labor	36.4 (0.71)	37.2 (0.75)		24.1 (2.04)	23.8 (3.64)	
None	23.2 (0.58)	18.3 (0.61)		59.5 (2.40)	47.7 (4.60)	
**Residence** (% rural)	17.3 (0.88)	17.8 (0.98)	0.5173	22.0 (2.05)	23.5 (3.76)	0.7086
**Household income** (quartiles)			<0.0001			<0.0001
1st (lowest)	21.8 (0.57)	26.7 (0.70)		22.2 (1.91)	41.4 (4.27)	
2nd	25.3 (0.59)	26.1 (0.67)		25.3 (2.09)	23.3 (3.67)	
3rd	25.5 (0.57)	25.0 (0.67)		23.8 (2.03)	17.4 (3.12)	
4th (highest)	27.5 (0.66)	22.2 (0.69)		28.7 (2.10)	17.9 (3.24)	
Lifetime smoking amount (pack-years), mean ± SE	18.08 ± 0.22	17.51 ± 0.22	0.0529	28.21 ± 1.09	25.02 ± 1.17	0.0439
**Secondhand smoke exposure**						
Workplace (yes)	30.1 (0.66)	41.3 (0.76)	<0.0001	14.0 (1.74)	23.0 (4.18)	0.0269
Home (yes)	2.4 (0.21)	7.6 (0.44)	<0.0001	4.0 (1.11)	2.8 (1.09)	0.4577
**Smoking cessation methods**						
Willpower	93.6 (0.32)	84.4 (0.52)	<0.0001	94.9 (1.06)	88.3 (2.65)	0.0066
Nicotine replacement therapy	6.0 (0.32)	17.3 (0.55)	<0.0001	4.9 (1.08)	10.0 (2.53)	0.0347
Education/counselling	5.2 (0.29)	11.9 (0.45)	<0.0001	4.0 (0.84)	12.9 (2.69)	<0.0001
Smokers’ quitline	0.6 (0.10)	1.4 (0.16)	<0.0001	0.3 (0.22)	1.4 (0.85)	0.0669

Data are presented as weighted mean ± standard error (SE) or weighted percent (SE). The p-values were determined by Student's t-test or Rao-Scott chi-squared test. Income quartiles are age and gender adjusted. CKD: chronic kidney disease.

**Table 3:** Clinical characteristics, health behaviors, and perceived health (N=15747)

*Variables*	*Non-CKD (n=14838)*			*CKD (n=909)*		
	*Quitter (n=8393) % (SE)*	*Non-quitter (n=6445) % (SE)*	*p*	*Quitter (n=703) % (SE)*	*Non-quitter (n=206) % (SE)*	*p*
**Health status**						
eGFR, mean ± SE	86.16 ± 0.20	90.94 ± 0.23	<0.0001	50.41 ± 0.53	50.12 ± 0.98	0.7943
eGFR ≥90	35.9 (0.68)	49.7 (0.78)	<0.0001	-	-	0.4441
eGFR 89–60	64.1 (0.68)	50.3 (0.78)		-	-	
CKD Stage 3^a^	-	-		79.6 (1.91)	77.2 (3.50)	
CKD Stage 3^b^	-	-		15.3 (1.66)	14.7 (2.90)	
CKD Stage 4–5	-	-		5.2 (1.14)	8.1 (2.36)	
Systolic BP (mmHg), mean ± SE	121.36 ± 0.21	117.86 ± 0.22	<0.0001	127.16 ± 0.80	126.95 ± 1.59	0.9075
Diastolic BP (mmHg), mean ± SE	78.86 ± 0.15	78.03 ± 0.17	<0.0001	74.97 ± 0.55	76.49 ± 1.21	0.2524
Body mass index (kg/m^2^), mean ± SE	24.52 ± 0.04	24.27 ± 0.05	<0.0001	24.52 ± 0.13	24.47 ± 0.32	0.8899
Waist circumference (cm), mean ± SE	86.42 ± 0.12	85.07 ± 0.14	<0.0001	88.30 ± 0.41	88.00 ± 0.83	0.7511
Fasting blood sugar (mg/dL), mean ± SE	103.00 ± 0.29	99.62 ± 0.35	<0.0001	109.67 ± 1.33	112.37 ± 3.43	0.4654
Total cholesterol (mg/dL), mean ± SE	190.46 ± 0.49	190.46 ± 0.55	0.9901	177.97 ± 1.75	182.41 ± 4.26	0.3449
Hypertension	35.9 (0.62)	23.3 (0.63)	<0.0001	74.5 (2.04)	73.3 (3.81)	0.7895
Diabetes mellitus	13.0 (0.42)	9.3 (0.40)	<0.0001	31.5 (2.14)	38.5 (4.41)	0.1417
Cardiovascular disease	5.6 (0.26)	2.6 (0.20)	<0.0001	23.8 (1.96)	12.2 (2.53)	0.0012
Cancer	4.3 (0.23)	1.2 (0.14)	<0.0001	9.1 (1.28)	3.8 (1.46)	0.0243
Trauma history	7.2 (0.36)	9.6 (0.45)	<0.0001	5.4 (1.12)	7.2 (2.01)	0.4291
Heavy drinking	19.7 (0.54)	29.3 (0.67)	<0.0001	6.4 (1.17)	9.2 (2.34)	0.2420
Regular exercise	41.8 (0.70)	37.7 (0.74)	<0.0001	30.5 (2.16)	20.7 (3.74)	0.0403
Sleep duration (h/day), mean ± SE	6.96 ± 0.02	6.98 ± 0.02	0.4065	7.06 ± 0.09	7.09 ± 0.12	0.8553
**Skipping meals**						
Breakfast	16.9 (0.55)	32.5 (0.73)	<0.0001	6.9 (1.31)	13.5 (3.33)	0.0280
Lunch	5.5 (0.31)	9.3 (0.47)	<0.0001	5.5 (1.02)	10.0 (2.71)	0.0647
Dinner	3.3 (0.24)	5.2 (0.32)	<0.0001	4.1 (1.11)	2.1 (1.04)	0.2319
**Eating out** (frequency)			<0.0001			0.2208
≥Once/day	35.2 (0.70)	42.7 (0.80)		11.6 (1.61)	15.0 (3.34)	
1–6 time(s)/week	43.4 (0.68)	43.0 (0.77)		42.1 (2.33)	35.3 (4.14)	
1–3 time(s)/month	14.9 (0.45)	10.0 (0.43)		27.9 (2.00)	34.5 (4.30)	
<Once/month	6.5 (0.29)	4.2 (0.26)		18.4 (1.70)	15.3 (2.72)	
**Drinking coffee** (frequency)			0.0071			0.0294
≥Once/day	71.4 (1.40)	77.2 (1.30)		52.6 (8.85)	81.9 (8.10)	
1–6 time(s)/week	16.1 (1.12)	13.1 (1.02)		30.8 (8.81)	14.8 (7.85)	
0–3 time(s)/month	12.5 (1.00)	9.7 (0.87)		16.6 (6.32)	3.3 (2.50)	
**Perceived health status**			<0.0001			0.2381
Very good/good	39.0 (0.64)	32.6 (0.70)		24.0 (1.90)	18.7 (3.64)	
Fair	46.6 (0.65)	51.2 (0.74)		43.0 (2.31)	40.7 (4.49)	
Poor/very poor	14.4 (0.45)	16.3 (0.54)		33.1 (2.19)	40.7 (4.43)	
Perceived psychological stress*	22.0 (0.57)	31.8 (0.68)	<0.0001	11.6 (1.49)	27.3 (4.20)	<0.0001

Values are presented as sample n, weighted mean ± standard error (SE) or weighted percentage (SE): The p values were determined by Student's t-test or Rao–Scott chi-squared test . CKD Stage 3a = eGFR 45–59. CKD Stage 3b = eGFR 30–44. CKD Stage 4–5 = eGFR <30. Drinking coffee was surveyed from 2012–2016 only for the ages of 19–64 years.

* The question was: ‘How stressed are you on a daily basis?’.

The survey participants responded by selecting one of the following responses: ‘extremely stressed (severe)’, ‘quite stressed (moderate)’, ‘a little bit stressed (mild)’, or ‘not stressed at all (none)’. In this study, perceived psychological stress was defined as the percentage of participants with severe or moderate stress among all participants. BP: blood pressure. CKD: chronic kidney disease. eGFR: estimated glomerular filtration rate.

Variables with p<0.1 in Tables 2 and 3 were used for the multiple logistic regression. Wald test with multiple logistic regression analyses was performed to investigate factors associated with smoking cessation in both CKD and non-CKD groups and the interaction effects between the groups. All estimates for all interaction terms between CKD and non-CKD groups were obtained from the multiple logistic regression adjusting for the involved characteristics such as age, marital status, household income, lifetime smoking amount, secondhand smoke exposure, and smoking cessation methods. Diastolic blood pressure and waist circumference were omitted from the analysis because of multi-collinearity. Drinking coffee was also omitted because it was surveyed only from 2012–2016. A p<0.05 or 95% confidence interval (CI) that did not span 1.0 was considered statistically significant (Table 4). As KNHANES’ data are derived from stratified and multistage clustered probability sampling methods to represent the entire Korean population, population weights were applied to the analyses^31^.

**Table 4:** Factors associated with quitting smoking in non-CKD and CKD groups

*Variables*	*Non-CKD AOR (95% CI)*	*CKD AOR (95% CI)*	Interaction p*
**Age** (years)			0.5481
19–39 (Ref.)	1	1	
40–64	2.07 (1.83–2.34)	1.20 (0.21–6.73)	
≥65	4.82 (3.99–5.82)	2.27 (0.41–12.57)	
**Marital status** (married)	2.07 (1.82–2.35)	0.86 (0.43–1.71)	0.0139
**Occupation**			0.1587
Manager/professionals (Ref.)	1	1	
Office worker	1.01 (0.85–1.19)	6.10 (1.48–25.11)	
Service workers/sellers	0.86 (0.72–1.02)	1.67 (0.45–6.22)	
Agriculture/fishery/labor	0.94 (0.81–1.09)	2.46 (0.85–7.13)	
None	0.94 (0.81–1.09)	2.52 (0.97–6.54)	
**Household income** (% in quartiles)			0.1743
1st (lowest) (Ref.)	1	1	
2nd	1.14 (1.00–1.30)	1.76 (0.92–3.38)	
3rd	1.16 (1.02–1.33)	2.04 (1.05–3.96)	
4th (highest)	1.33 (1.16–1.53)	2.67 (1.32–5.42)	
**Lifetime smoking amount** (each 10 pack-year increase)	0.86 (0.83–0.88)	1.06 (0.97–1.16)	<0.0001
**Secondhand smoke exposure**			
Workplace (yes)	0.84 (0.76–0.94)	0.79 (0.38–1.66)	0.8592
Home (yes)	0.48 (0.37–0.63)	1.91 (0.53–6.91)	0.0396
**Smoking cessation methods**			
Willpower	1.53 (1.28–1.83)	1.58 (0.63–3.93)	0.9509
Nicotine replacement therapy	0.46 (0.38–0.55)	0.57 (0.22–1.50)	0.6617
Education/counselling	0.55 (0.46–0.66)	0.41 (0.17–0.99)	0.5304
Smokers’ quitline	0.74 (0.45–1.21)	0.56 (0.10–3.16)	0.7619
**Health status**			
eGFR, per 10-unit increase	0.91 (0.88–0.95)	0.91 (0.70–1.17)	0.9472
Systolic blood pressure (each 10-mmHg increase)	1.07 (1.02–1.11)	0.89 (0.79–1.01)	0.0067
Body mass index (kg/m^2^)	1.04 (1.02–1.06)	1.07 (0.98–1.17)	0.5297
Fasting blood sugar (each 10 mg/dL increase)	1.03 (1.00–1.05)	0.99 (0.92–1.07)	0.3579
Hypertension	1.19 (1.05–1.34)	1.09 (0.64–1.86)	0.7579
Diabetes mellitus	0.86 (0.71–1.04)	0.79 (0.46–1.35)	0.7560
Cardiovascular disease	1.22 (0.97–1.53)	1.96 (1.04–3.69)	0.1666
Cancer	1.98 (1.47–2.65)	1.95 (0.72–5.23)	0.9765
Trauma history	0.84 (0.71–1.00)	1.57 (0.60–4.15)	0.2150
Heavy drinker	0.68 (0.61–0.76)	1.17 (0.49–2.83)	0.2279
Regular exercise	1.29 (1.17–1.42)	2.84 (1.52–5.31)	0.0153
**Skipping meals**			
Breakfast	0.64 (0.57–0.73)	0.62 (0.27–1.46)	0.9427
Lunch	0.68 (0.56–0.82)	0.41 (0.14–1.22)	0.3758
Dinner	0.78 (0.63–0.98)	3.23 (0.68–15.28)	0.0754
**Eating out** (frequency)			0.1211
≥Once/day (Ref.)	1	1	
1–6 time(s)/week	1.02 (0.92–1.14)	1.30 (0.56–2.98)	
1–3 time(s)/month	1.08 (0.92–1.27)	0.63 (0.27–1.45)	
<Once/month	0.93 (0.75–1.15)	0.76 (0.33–1.78)	
**Perceived health status**			0.9139
Very good/good	1.44 (1.24–1.66)	1.24 (0.61–2.55)	
Fair	1.12 (0.98–1.28)	1.02 (0.59–1.79)	
Poor/very poor (Ref.)	1	1	
Perceived psychological stress	0.79 (0.71–0.88)	0.46 (0.25–0.87)	0.1061

* p-values were obtained by the Wald test with logistic regression.

CI: confidence interval. CKD: chronic kidney disease. eGFR: estimated glomerular filtration rate. AOR: adjusted odds ratio.

## RESULTS

### Prevalence of quitters and non-quitters

Table 1 displays the prevalence rates of quitters and non-quitters of the non-CKD and the CKD groups from 2008 to 2019, excluding 2013. Among the 15747 participants, 909 individuals suffered from CKD. Of the 14838 individuals in the non-CKD group, 8393 (weighted percentage: 50.4%) succeeded in quitting. Among the 909 individuals in the CKD group, 703 (weighted percentage: 74.4%) were quitters. In the non-CKD group, the success rates of smoking cessation tended to increase during the study period. The success rates showed slight fluctuations over the study period in the CKD group but were generally steady. The success rates of the CKD group were consistently higher than those in the non-CKD group throughout the study period.

### Sociodemographic characteristics and smoking history

Table 2 shows the participants’ sociodemographic characteristics and smoking history. In the non-CKD group, quitters were more likely to be older, married, and have higher incomes than non-quitters. Secondhand smoke exposure either at the workplace or at home was more common in non-quitters. As for the smoking cessation method, quitters used willpower more often, whereas non-quitters chose nicotine replacement therapy (NRT), education/counselling, and quitlines more frequently.

In the CKD group, quitters were more likely to be older and have higher incomes. The lifetime smoking amount in quitters was 28.2 pack-years, significantly higher than the 25.0 pack-years in non-quitters (p=0.0439). Secondhand smoke exposure at the workplace was more common in non-quitters. Quitters used willpower more often regarding the smoking cessation method, whereas non-quitters chose NRT and education/counselling more frequently.

### Clinical characteristics and health behaviors and perceptions

Table 3 displays the participants’ clinical characteristics, health behaviors, and perceptions, for both groups. In the non-CKD group, blood pressure, body mass index, waist circumference, and fasting blood sugar were higher in quitters. Comorbidities such as hypertension, diabetes mellitus, CVD, and cancer prevalence were more often observed in quitters. Quitters exercised regularly and more frequently. In contrast, trauma history, heavy drinking, skipping meals (breakfast, lunch, or dinner), eating out, and drinking coffee more than once per day, poor perceived health status, and high perceived psychological stress were more often observed in non-quitters.

CVD, cancer prevalence, and regular exercise were more frequently observed in the CKD group in quitters. Skipping breakfast, drinking coffee more than once per day, and high perceived psychological stress were more common among non-quitters.

### Factors associated with smoking cessation

**Figure 2:**
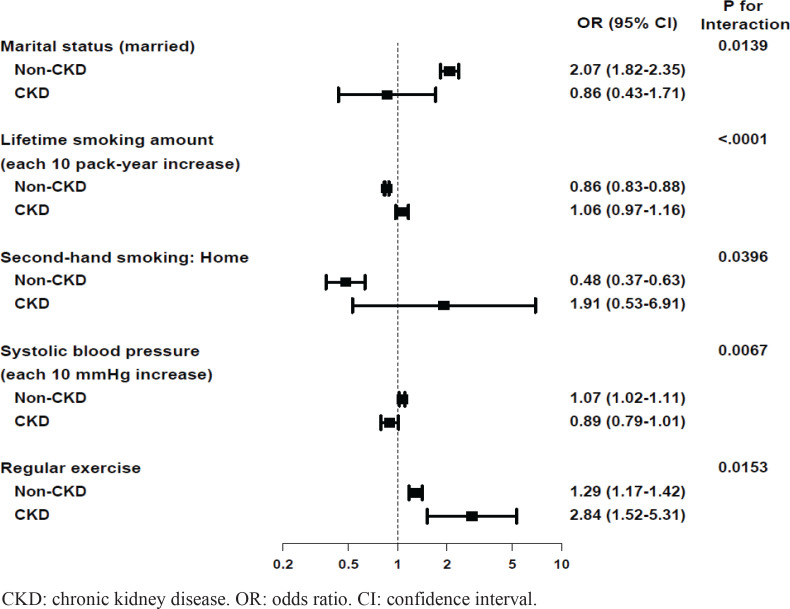
Significant factors associated with quitting smoking in non-CKD and CKD groups with p for the interactions between two groups

Table 4 shows the factors associated with quitting smoking and the interaction effects between the non-CKD and CKD groups. Additionally, Figure 2 shows the significant factors and p-values for the interactions between the two groups.

Regular exercise was the only factor that was significantly associated with quitting in both non-CKD and CKD groups, indicating that people who exercise regularly have a 1.29-fold and 2.84-fold increase in quitting success rate in the non-CKD and the CKD groups, respectively (adjusted odds ratio, AOR=1.29; 95% CI: 1.17–1.42 for the non-CKD group; AOR=2.84; 95% CI: 1.52–5.31 for the CKD group, interaction p=0.0153). Unlike in the CKD group, a few more variables were also associated with quitting in the non-CKD group. Marriage and higher systolic blood pressure (SBP) were associated with quitting only in the non-CKD group (marriage: AOR=2.07; 95% CI: 1.84–2.39, interaction p=0.0139; SBP: AOR=1.07; 95% CI: 1.02–1.11, interaction p=0.0067), while lifetime smoking amount and secondhand smoke exposure at home were negatively associated with quitting smoking in the non-CKD group (lifetime smoking amount: AOR=0.86; 95% CI: 0.83–0.88, interaction p<0.0001; secondhand smoke exposure at home: AOR=0.48; 95% CI: 0.37–0.63, interaction p=0.0396).

## DISCUSSION

This study aimed to examine and compare factors associated with quitting smoking in Korean adult male smokers, with and without CKD, using KNHANES data. The findings can be summarized as follows: 1) smoking cessation rates in the CKD group remained relatively stable during the study period, whereas those in the non-CKD group tended to increase; 2) fewer factors were significant in quitting smoking in the CKD group than in the non-CKD group; and 3) the interaction effects between the two groups revealed that regular exercise was the only factor associated with quitting in the CKD group.

As stated above, smoking cessation rates in participants without CKD tended to increase over the study period; this has been previously observed in Korean male smokers (Table 1)^30^. During the study period, cessation rates in the CKD group did not seem to increase but were higher than those in the non-CKD group throughout the study. It has been reported that smokers with chronic medical conditions attempt to quit smoking and use evidence-based tobacco-cessation treatment more frequently than smokers without comorbidities. Smoking in individuals with chronic medical conditions can negatively affect symptoms, disease progression, and mortality. Therefore, chronic comorbidities can often motivate quitting smoking, but it is controversial whether this motivation leads to quitting^30,32,33^. However, our study findings suggest that CKD may have motivated patients to quit. Another possibility is that older age is associated with a higher success rate of quitting smoking among Korean adult males, as has been reported from previous national surveys. Given that the CKD group is older than the non-CKD group, this age difference might explain the higher smoking cessation rate in the CKD group^30,33,34^.

In the CKD group, significant differences were observed in fewer clinical characteristics between quitter and non-quitters than in the non-CKD group (Tables 2 and 3). For instance, among quitters in the non-CKD group, most comorbid conditions were common, but only cardiovascular comorbidity and cancer were frequently observed among those in the CKD group. This may be simply because of the higher prevalence of CVD and cancer in patients with CKD than in the general population, as they are the leading cause of death in the CKD population^1,3,4,35^. However, the explanation of this finding, i.e. fewer factors associated with quitting in the CKD group, remains unclear. It has been reported that patients with CKD tend to share many commonalities in socioeconomic status, demographic characteristics, health perception and behavior, and comorbidities, which might explain the small number of differences between two groups (quitter vs non-quitter) in patients with CKD^3^.

When the interaction effects between groups were evaluated, regular exercise was the only factor associated with cessation in the CKD group (Table 4 and Figure 2). Exercise was associated with quitting in the non-CKD group as well; however, based on the AOR, exercise had a more significant effect on smoking cessation in the CKD than in the non-CKD group. Despite extensive research on the importance of exercise in smoking cessation, there does not seem to be a consensus on its effect^36-38^. Regular exercise is expected to contribute to smoking cessation by attenuating cigarette withdrawal symptoms and cravings or controlling weight^36,37^. A systematic review suggests that aerobic exercise may be effective in quitting smoking in the short-term, but not in the medium-term to long-term follow-up^37^. However, another review reports no effect of exercise on smoking cessation, regardless of the type of exercise^38^.

In the non-CKD group, the interaction effects revealed the associations of several other variables with smoking cessation. Marriage and SBP were associated with quitting, while secondhand smoke exposure and greater lifetime smoking were negatively associated with quitting. Previous national surveys have reported older age, being married, higher education level, using willpower to quit, alcohol abstinence, smoke-free environment, or better stress management, as successful smoking cessation factors in Korean adult males. Therefore, our study findings are consistent with existing national surveys investigating smoking cessation success factors in Korean male adults^30,33,34^.

This study has examined a wide range of potential predictors of quitting smoking, such as ‘skipping meals’ or ‘frequency of eating out’. We selected these variables as potential predictors for the following reasons: 1) the study aim was to find the disease-specific factors associated with quitting status. CKD is a disease in which dietary precautions are critical to modify disease progression. It is recommended for patients with CKD to eat regularly and dine out less frequently^39^; 2) ‘meal skipping’ or ‘frequent eating out’ were previously reported as predictors of smoking cessation failure^30^. Viewed in combination, we speculated that these variables could represent overall treatment adherence, including smoking cessation, in patients with CKD. Therefore, we included these variables in the analysis.

### Limitations

There are a few limitations to this study. The reliability of smoking history may be lower because the survey was conducted only with self-report. Although we analyzed a comprehensive set of potential predictors of smoking cessation, we did not include some important, evidence-based predictors in the analysis. For example, the analysis did not include levels of tobacco dependence and motivation to quit smoking, history of using e-cigarettes or history of mental disorders such as anxiety or depression. The reason for this was that the KNHANES does not provide such information. In particular, people with mental illnesses tend to be more heavily addicted to nicotine when they smoke, have greater difficulty quitting, but are less likely to get help with quitting than the general population of smokers. Regrettably, mental illness could not be analyzed in this study^40^.

Notably, a diagnosis of CKD requires maintaining chronicity of reduced eGFR for at least three months However, as the KNHANES is a cross-sectional survey, we could not confirm the chronicity of kidney dysfunction in the study participants. Therefore, some participants with acute kidney injury might have been included in the CKD group^26,27^.

Lastly, our study adopted a cross-sectional design with the associated limitations in interpreting the results. Thus, further studies should address the causal relationship between exercise and smoking cessation in the population with CKD to examine the replicability of our study findings, particularly since no previous studies have examined disease-specific smoking cessation factors.

## CONCLUSIONS

Exercise was the only factor associated with quitting smoking in the CKD group. Further analysis using a longitudinal design to determine the role of exercise in smoking cessation in the CKD population will be needed. This study showed that participants with CKD differed in factors associated with quitting smoking compared to participants without CKD. This may imply a need for disease-specific approach to improving the effectiveness of smoking cessation.

## Data Availability

The data supporting this research are available from the following sources: http://knhanes.cdc.go.kr/
